# The structure of FKBP38 in complex with the MEEVD tetratricopeptide binding-motif of Hsp90

**DOI:** 10.1371/journal.pone.0173543

**Published:** 2017-03-09

**Authors:** Katie L. I. M. Blundell, Mohinder Pal, S. Mark Roe, Laurence H. Pearl, Chrisostomos Prodromou

**Affiliations:** Genome Damage and Stability Centre, School of Life Sciences, University of Sussex, Falmer, Brighton BN1 9RQ, England; Consiglio Nazionale delle Ricerche, ITALY

## Abstract

Tetratricopeptide (TPR) domains are known protein interaction domains. We show that the TPR domain of FKBP8 selectively binds Hsp90, and interactions upstream of the conserved MEEVD motif are critical for tight binding. In contrast FKBP8 failed to bind intact Hsp70. The PPIase domain was not essential for the interaction with Hsp90 and binding was completely encompassed by the TPR domain alone. The conformation adopted by Hsp90 peptides, containing the conserved MEEVD motif, in the crystal structure were similar to that seen for the TPR domains of CHIP, AIP and Tah1. The carboxylate clamp interactions with bound Hsp90 peptide were a critical component of the interaction and mutation of Lys 307, involved in the carboxylate clamp, completely disrupted the interaction with Hsp90. FKBP8 binding to Hsp90 did not substantially influence its ATPase activity.

## Introduction

**Fig 1:**
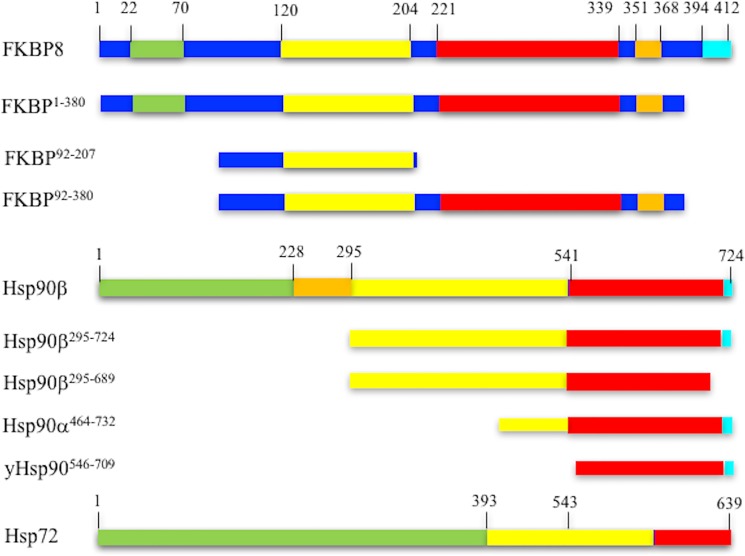
Constructs and domain boundaries. FKBP8: green, glutamate rich domain; yellow, PPIase domain; red, TPR domain; orange, calmodulin binding region and cyan, transmembrane region. Hsp90: green, N-terminal domain; orange, charged linker region; yellow, middle domain; red, C-terminal domain and cyan, conserved MEEVD TPR-binding motif. Hsp72: green, N-terminal domain; yellow, substrate binding domain and red, lid region. Numbers represent the approximate domain boundaries.

Immunophilins are a highly-conserved family of proteins that bind immune-suppressive drugs such as cyclosporin, rapamycin and FK506 [1–3]. FKBP8 consists of an N-terminal glutamate rich region and a PPIase domain that is connected by a loop to a tetratricopeptide domain (TPR) (**Fig 1**). Downstream of the TPR domain is a calmodulin binding- and a membrane anchor-region at the extreme C-terminus (**Fig 1**). Two isoforms of this immunophilin have been described, the full-length FKBP8 and a product of the truncated ORF of this gene known as FKBP38 [4].

FKBP8 belongs to the FK506 family of binding proteins, but unlike many members of this family it lacks a constitutive PPIase activity, which instead relies on regulatory control [5, 6]. Studies show that the structural fold of FKBP8 is almost identical to that of FKBP12, except for a larger loop structure between β-strand C and D of FKBP12 [7, 8]. The lack of a constitutive PPIase activity in FKBP8 has been attributed to the side chain of Arg 184 (donated as Arg 127 in [8]), which occludes access to the active site of the catalytic domain [8]. FKBP8 is unique amongst typical PPIases in that its PPIase activity is regulated by the calcium sensor calmodulin (CaM), by interaction with the PPIase domain of FKBP8, including the N-terminal glutamate rich region and with a second site downstream of the TPR domain of FKBP8 [9, 10]. The regulation of FKBP8 is further complicated by the presence of a charge-sensitive loop near the putative active site, which acts as a cation coordination site involving two aspartic acid residues. However, the precise details of the activation of FKBP8 remain unknown. In contrast to FKBP8, FKBP52 consists of two immunophilin domains, and crystallization studies show that only the N-terminal of the two immunophilin domains is occupied by FK506 [7]. It appears that the C-terminal immunophilin domain possess a helical element, formed by a longer amino acid sequence in this region of the domain (DLGKGEVI versus EIGEGENLDLP), that apparently occludes FK506 binding.

Activated FKBP8 has been shown to bind Bcl-2 and block its interaction with the pro-apoptotic protein, Bad [11], an activity which is inhibited by the molecular chaperone Hsp90 [12, 13]. Beyond apoptosis, FKBP8 has also been shown to play a role in neural tube formation, regulation of mTOR kinase activity and regulation of cellular hypoxia and Hepatitis C virus replication (HCV) [14–21]. It’s role in virus replication appears to be Hsp90 dependent [18]. Human butyrate-induced transcript 1 (hB-ind1) has been found in complex with FKBP8 and Hsp90 and appears to be required for HCV replication [20, 21]. Finally, NS5A interacts with FKBP8 and can disrupt FKBP8-mediated mTOR regulation, while also stabilizing interaction with Hsp90 [11].

The TPR domain of FKBP8 is homologous to other such domains including AIP, CHIP, HOP and Tah1p [22–24]. These domains represent a protein interacting cleft that has been shown to bind either Hsp90 (TPR-1 domain of HOP and Tah1p), Hsp70 (TPR-2A domain of HOP), or more extensively the same TPR domain has been shown to bind both Hsp90 and Hsp70 (CHIP) or Hsp90, Hsp70 and TOMM20 (AIP) [24–27]. This study aims to address the specificity of the FKBP8 TPR domain towards Hsp90 and Hsp70 using structural and biochemical studies.

## Materials and method

### Protein expression and purification

FKBP8 (FKBP^1-380^) was cloned as an NdeI-HindIII fragment representing amino acids 1–380 and lacking the C-terminal trans-membrane helix into pDMXV4 with a non-cleavable C-terminal His_6_-Tag (**Fig 1**). The FKBP38 immunophilin domain, representing amino acids 92–207 (FKBP^92-207^), and a larger fragment containing both the immunophilin and TPR domain (FKBP^92-380^) were also cloned in pDMXV4 (**Fig 1**). The K307E mutation was generated in the FKBP8^92-380^ construct by PCR mutagenesis. Full-length human Hsp90β, human Hsp70, two middle domain human fragments (Hsp90β^295–689^ and Hsp90β^295–724^) and a yeast C-terminal fragment of Hsp90 (yHsp90^546-709^) were expressed from pRSETA as His-tagged fusions. The Hsp90α^464–732^ construct was expressed from p2E (A. Oliver, University of Sussex), as a PreScission cleavable His-tagged fusion. Proteins were overexpressed in *E*.*coli* BL21 (DE3) and purified by Talon affinity chromatography (Clontech, Oxford, England) equilibrated in 20 mM Tris pH 7.5 containing 100 mM NaCl and eluted with the same buffer but containing 500 mM imidazole at pH 7.0. Protein was then concentrated using Vivaspin concentrators (5,000 to 10,000 Da molecular-weight cutoff) and subjected, as appropriate, to Superdex 75, 200 or Sephacryl 300 HR gel-filtration chromatography equilibrated in 20 mM Tris pH 7.5 containing 500 mM NaCl, 1.0 mM EDTA and 0.5 mM TCEP. Proteins requiring further purification were subjected to Q-sepharose ion-exchange chromatography equilibrated in 20 mM Tris pH 7.5, 1 mM EDTA and 0.5 mM TCEP and eluted with a NaCl gradient in the same buffer. Pure protein was desalted in 20 mM Tris pH 7.5, 140 mM NaCl, 0.5 mM TCEP, concentrated and then stored frozen at −20°C.

### Analytical gel-filtration

Equimolar concentrations of the C-terminal fragment of yHsp90^546-709^ and FKBP^92-380^ were mixed and loaded onto an analytical Superdex 200 column equilibrated in 50 mM Tris, 300 mM NaCl, 1 mM EDTA and 0.5 mM TCEP at pH 7.5.

### Isothermal titration calorimetry

Proteins were dialysed overnight into 20 mM Tris, 300 mM NaCl, 1mM EDTA pH 7.5. The heat of interaction was measured on an ITC_200_ microcalorimeter (Microcal), with a cell volume of 200 μL, under the same buffer conditions. Typically, aliquots of 700 to 800 μM of FKBP^1-380^, FKBP^92-380^, FKBP^92-380^ K370E mutant protein, FKBP^92-207^ were injected into 50 μM of intact Hsp90β, Hsp90β^295–689^, Hsp90β^295–724^, Hsp90α^464–732^ or yeast Hsp90^546-709^. For peptide binding experiments aliquots of 2 mM of Hsp90β (EDASRMEEVD) or Hsp70 (GSGPTIEEVD) peptide were injected into 50 μM FKBP^1-380^. Heats of dilution were determined in a separate experiment by diluting protein into buffer, and the corrected data were fitted using a non-linear least-squares curve-fitting algorithm (Microcal Origin) with three floating variables: stoichiometry, binding constant and change in enthalpy of interaction.

### X-ray crystallography

Crystals of FKBP^92-380^ in complex with yHsp90 DTEMEEVD peptide were obtained by mixing FKBP^92-380^ with yHsp90^546-709^ in a 1:1 molar ratio at 18 mg ml^-1^ and crystalizing by using the sitting drop vapour diffusion technique. Crystals appeared in 0.02 M sodium potassium phosphate, 20% w/v PEG 3350 at 14°C. Data was collected from a single crystal to 2.2Å at the Diamond Synchrotron on beamline IO4_1 at wavelength 0.917Å. The dataset was processed using the DIALS [28]. Molecular replacement by Phaser (Phoenix) [29], was conducted with the PDB files 2AWG (FKBP^90-205,^ lacking the TPR domain) and 2FBN (plasmodium falciparum construct of a PPIase-TPR domain). Arp/Warp [30] and Buccaneer [31] were then used to build into the electron density. The structure then underwent several rounds of refinement in the Phoenix suite and manual building in Coot [32]. Final refinement was with Buster [33]. Crystallization data and statistics are given in **Table 1**. Structures and cartoons were displayed using PyMOl [34] and LigPlot [35, 36].

**Table 1:** Data collection and refinement statistics.

**Data Collection**	FKBP^92-380^-Hsp90 MEEVD
Wavelength (Å)	0.91741
Space group	P1 2_1_ 1
Unit cell a, b, c (Å)	74.29, 105.64, 100.19
*α*, *β*, *γ* ^(o)^	90.0, 93.1, 90.0
Resolution range (Å)	100–2.18 (2.24–2.18)
Total reflections	148001 (14436)
Unique reflections	76276 (3524)
Multiplicity	2.9 (2.9)
Completeness (%)	95.9 (96.0)
Mean I/σ(I)	5.0 (1.5)
Wilson β-factor	19.43
R_merge_ (%)	0.111 (0.492)
R_meas_ (%)	0.156 (0.694)
R_pim_ (%)	0.110 (0.489)
CC_1/2_	0.947 (0.733)
CC*	0.993 (0.722)
**Refinement**	
Reflections used in refinement	76276 (5509)
Reflections used for R-free	3524 (275)
R-work (%)	0.246 (0.330)
R-free (%)	0.308 (0.364)
Number of non-hydrogen bonds	9220
macromolecules	8162
Solvent	1058
Protein residues	1088
RMS (bonds) (Å)	0.009
RMS (angles) ^(o)^	1.15
Ramachandran favored, allowed, outliners (%)	96.46, 3.08, 0.47
Rotamer outliers (%)	3.26
Clashscore	3.31
Average B-factor (Å^2^)	33.57
macromolecules	33.66
Solvent	32.85

Highest shell in parentheses

## Results

### FKBP8 selectively binds Hsp90 using additional contacts outside the MEEVD motif

**Fig 2:**
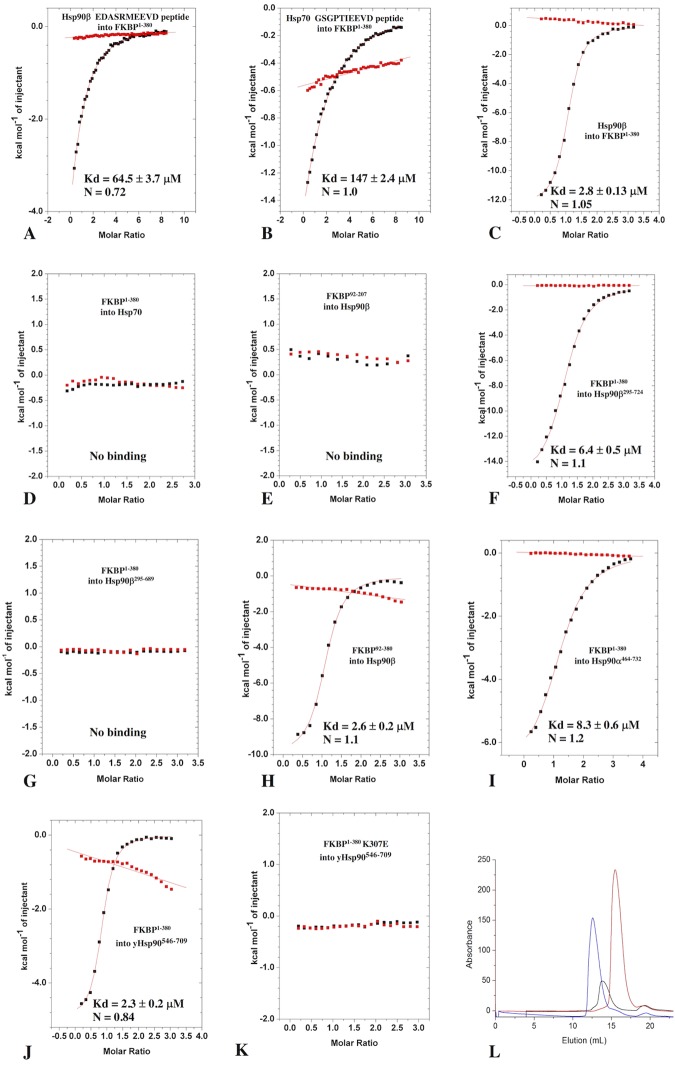
Isothermal titration calorimetry interactions. ITC interactions between FKBP^1-380^ and **A)**, Hsp90β EDASRMEEVD peptide **B)**; human Hsp70 GSGPTIEEVD peptide; **C),** full-length Hsp90β; **D)**, full-length human Hsp70; **E),** between FKBP^92-207^ and full-length Hsp90β; **F),** between FKBP^1-380^ and Hsp90β^295–724^ or **G),** Hsp90β^295–689^; **H),** between FKBP^92-380^ and full-length Hsp90**β; I),** between FKBP^1-380^ and human Hsp90α^464–732^; J), between FKBP^1-380^ and yHsp90^546-709^; K), and between the FKBP^1-380^ K307E mutant and yHsp90^546-709^. **L),** Gel-filtration chromatography of yHsp90^546-709^ (black trace), FKBP^1-380^ (red trace) and yHsp90546-709—FKBP^1-380^ (blue trace), showing that yHsp90^546-709^ and FKBP^1-380^ co-migrate as a complex.

TPR domains are known peptide interaction sites [37]. While most are specific for a particular peptide sequence, such as the TPR 2A domain of HOP and Tah1 that bind the conserved MEEEVD motif of Hsp90 [22, 23] or the TPR1A domain of HOP that recognises the conserved IEEVD motif of Hsp70 [27], others, such as CHIP and AIP, can accommodate both MEEVD and IEEVD peptides [23, 38]. Using isothermal titration calorimetry (ITC) to characterise the binding of the Hsp90 EDASRMEEVD and Hsp70 GSGPTIEEVD C-terminal peptides to the FKBP8 isoform (FKBP^1-380^, but lacking the transmembrane domain), showed that both peptides bound weakly to FKBP^1-380^ (Kd of 64.5 ± 3.7 and 147 μ 2.4 μM, respectively) (**Fig 2A and 2B**). In contrast to C-terminal peptides, full-length Hsp90β tightly bound FKBP^1-380^ (Kd of 2.8 ± 0.1 μM), while no binding interaction was seen for human Hsp70, suggesting that FKBP^1-380^ selectively binds Hsp90β and that binding may involve additional interactions outside those of the Hsp90β EDASRMEEVD peptide (**Fig 2C and 2D**). The immunophilin domain alone (FKBP^92-270^) failed to bind full-length Hsp90β, indicating that it was not essential for binding to Hsp90β (**Fig 2E)**. Using fragments of Hsp90 (Hsp90^295-724^ and Hsp90^295-689^) representing the middle and C-terminal domain of Hsp90β showed that sequences downstream of residue 689, that include the conserved MEEVD sequence, were essential for the interaction (**Fig 2F and 2G**). Hsp90^295-724^ bound FKBP^1-380^ with a similar affinity (Kd = 6.4 ± 0.5 μM; **Fig 2F**) to that seen for intact Hsp90β 2.8 ± 0.1 μM; **Fig 2C**). The FKBP38 isoform (FKBP^92-380^) bound Hsp90β with similar affinity (Kd = 2.6 ± 0.2 μM; **Fig 2H**) to that seen with FKBP^1-380^ (Kd of 2.8 ± 0.1 μM; **Fig 2C**). The stoichiometry for binding full-length Hsp90β was 1:1 (2 FKBP molecules to 1 Hsp90 dimer) for both the FKBP8 (FKBP^1-380^) and FKBP38 (FKBP^92-380^) isoforms. Finally, we showed that FKBP^1-380^ bound the C-terminal domain of Hsp90α (Hsp90α^464–732^ Kd = 8.3± 0.6 μM; **Fig 2I**) and yeast (yHsp90^546-709^; Kd 2.3± 0.2 μM **Fig 2J**), with similar affinities. Analytical gel-filtration chromatography showed that the C-terminal domain of yHsp90^546-709^ co-migrated with FKBP^92-380^ (**Fig 2L**), thus confirming the ITC interaction data.

### Structural features of FKBP38 in complex with Hsp90 MEEVD peptide

#### Structural overview

**Fig 3:**
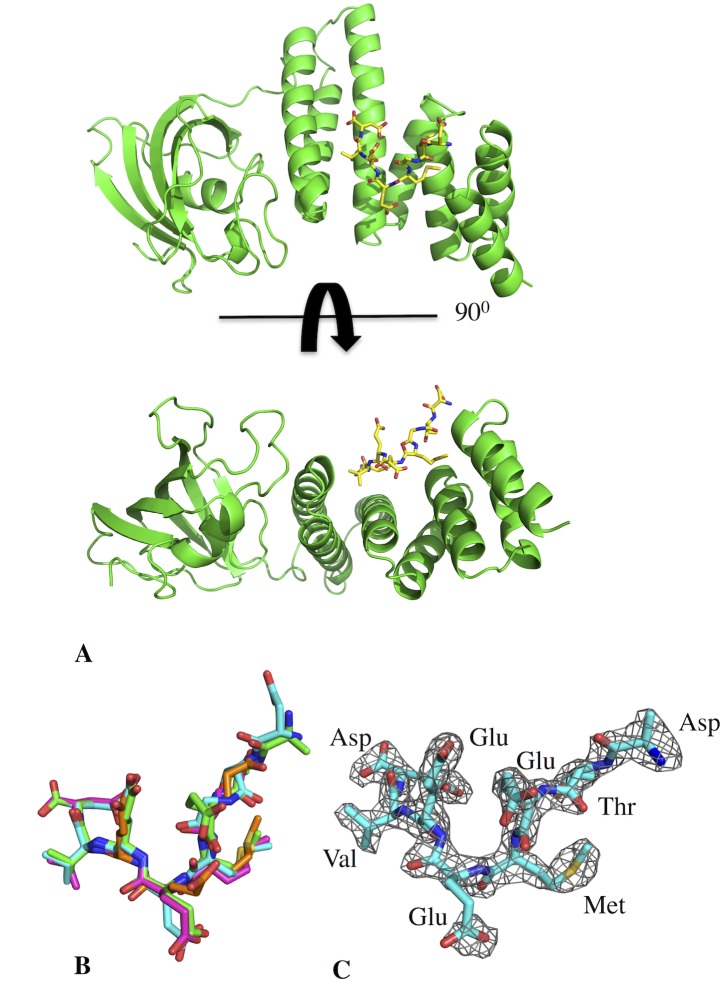
PyMol Cartoon structure of the FKBP^92-380^-DSTMEEVD complex. **A),** FKBP^92-380^ consists of a single PPIase domain (left domain) and a TPR domain (right helical domain) that acts as the binding site of the DSTMEEVD Hsp90β peptide (yellow stick representation). **B**), Superimposition of the MEEVD containing peptides found bound to the four TPR domains of FKBP^92-380^ of the unit cell of the crystal. The peptide sequences bound were DTEMEEVD, ATEMEEVD (where the side chain of the first aspartate residue was modeled as an alanine residue), EMEEVD and EMEE. For amino acid residues defined by visible density the conformation of the amino acid was very similar in all cases. **C),** Representation of the electron density shown as a mesh for the bound DTEMEEVD peptide.

Attempts to crystallise the C-terminal domain of yeast Hsp90 with FKBP^92-380^ yielded crystals of FKBP^92-380^ in complex with MEEVD containing peptides of the C-terminal domain of yeast Hsp90. (**Fig 3A and 3B**). It was clear that during crystallization the C-terminal domain of Hsp90 had undergone proteolytic cleavage to leave the C-terminal MEEVD peptides bound to the TPR domain of FKBP^92-380^. Four molecules of FKBP^92-380^ were observed in the unit cell of the crystal and the structure was solved at 2.18 Å (PDB 5MGX) resolution (**Table 1**). The bound peptides found were DTEMEEVD, ATEMEEVD (where the side chain of the first aspartate residue was modeled as an alanine), EMEEVD and EMEE. For amino acid residues defined by visible electron density the conformation of the amino acid at similar positions was very similar in all cases (**Fig 3B and 3C**).

**Fig 4:**
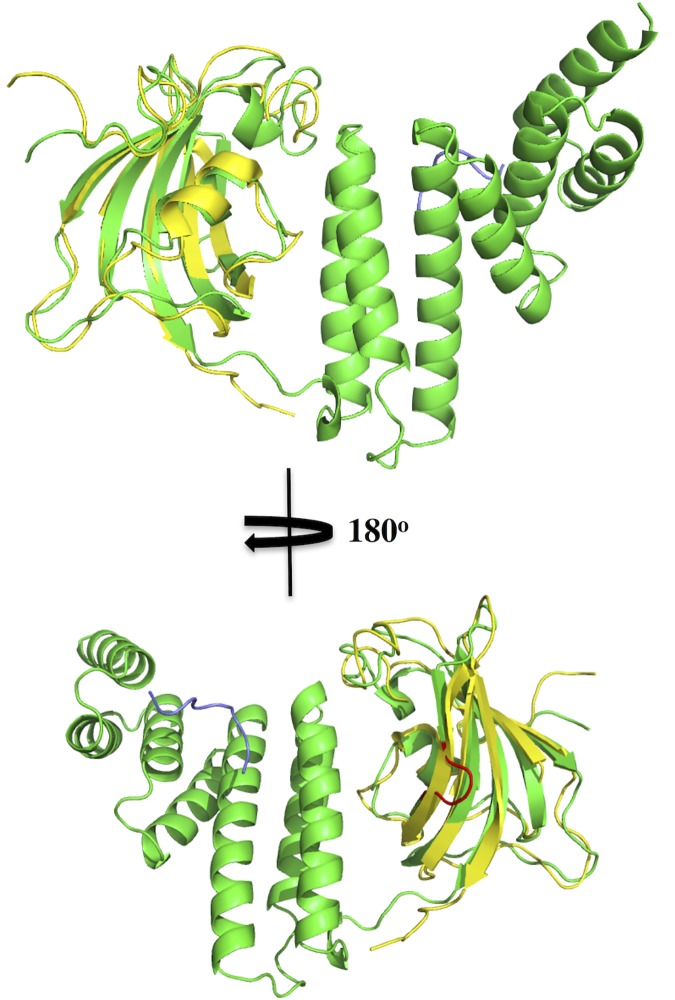
Superimposition with the NMR structure of the PPIase domain of FKBP38. Superimposition of the PPIase domains from NMR (yellow) and crystallography (green) show that the structures are essentially the same. However, in the crystal structure, relative to that of the NMR structure, a loop is present (Red) that disrupts β-strand 3 into two shorter β-strands.

**Fig 5:**
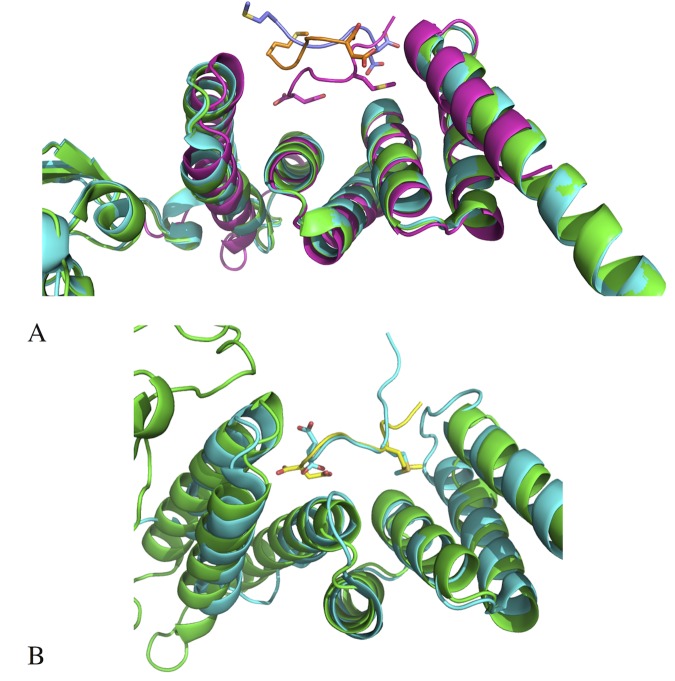
Superimposition of the TPR domain of FKBP52 and FKBP^92-380^. **A),** Superimposition of the TPR domains of two molecules of the unit cell of the FKBP52 structure with bound peptide (green colored molecule with gold peptide and cyan colored molecule with blue peptide) and that of FKBP^92-380^ (magenta colored molecule and peptide) showing that the TPR domains are essentially the same. However, the bound MEEVD containing peptides are in radically different conformations. The peptides from the FKBP52 structure do not superimpose and are bound in an opposite polarity to that seen for FKBP^92-380^. While the hydrophobic residues, methionine and valine, of the conserved MEEVD motif of Hsp90 are buried in hydrophobic pockets of FKBP^92-380^, the same residues are surprisingly exposed to solvent in the FKBP52 structure. **B),** Superimposition of FKBP^92-380^ (green molecule with yellow bound peptide) and the TPR domain of CHIP (cyan molecule and bound peptide) showing that the bound MEEVD containing peptide conformations are essentially the same.

**Fig 6:**
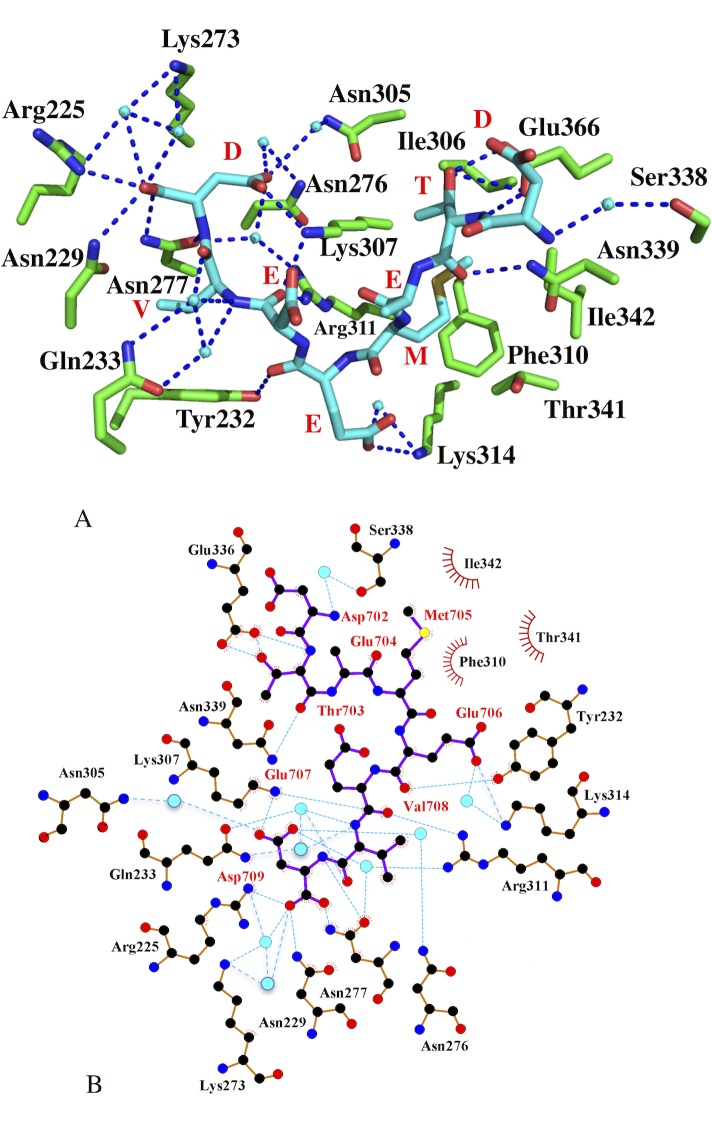
Illustrations of the bound DTEMEEVD peptide. **A),** Pymol cartoon showing interactions between the DTEMEEVD peptide (cyan) and the FKBP^92-380^ protein (green). Hydrogen bonds and salt bridges are shown as dotted blue lines and water molecules as cyan spheres. **B),** Ligplot showing the interactions between the DTEMEEVD peptide and the FKBP^92-380^ protein. Hydrogen bonds and salt bridges are shown as dotted cyan colored lines and water molecules as cyan spheres. Red amino acid residues represent the peptide and those in black represent FKBP^92-380.^

The immunophilin domain of FKBP^92-380^ is similar to that solved by NMR, except in the crystal structure of FKBP^92-380^ a section in the middle of β-strand 3 of the immunophilin domain is restructured into a loop (**Fig 4**). Superimposition of FKBP^92-380^ with FKBP52 [39] shows that the TPR domains are essentially the same consisting of three pairs of anti-parallel helices and a C-terminal α-7 helix (**Fig 5A**). However, the DTEMEEVD peptide of the TPR domain of FKBP^92-380^ is bound in an opposite polarity to that observed for the MEEVD peptide bound to FKBP52 (**Fig 5A**). In addition to the unexpected polarity, the conformation of the bound peptide for two molecules of FKBP52, found bound with peptide in the unit cell, was modelled differently (**Fig 5**A). On closer inspection, the methionine and valine residues of the bound MEEVD peptide are unexpectedly exposed to solvent in the FKBP52 structure (**Fig 5A**). In contrast, a comparison with the MEEVD binding TPR domain of CHIP [24] shows that the conformation of the Hsp90 peptide in both FKBP^92-380^ and CHIP is almost identical (**Fig 5B**), and therefore similar to those also seen in AIP and Tah1 [22, 23]. Because these structures share a common peptide polarity, and the hydrophobic methionine and the valine residues of the DTEMEEVD peptide are buried, as expected, in hydrophobic pockets, it is highly likely that this conformation represents the physiologically bound state for Hsp90 peptide (**Fig 6**). It is evident that the peptide bound to FKBP52 is most likely not bound in the physiologically relevant state, or was modelled incorrectly.

The side chain of Arg 184 (donated as Arg 127 in [8]) occludes access to the active site of the catalytic domain and therefore thought to be responsible for the lack of constitutive PPIase activity [8]. Instead PPIase activity is regulated by the calcium sensor calmodulin (CaM) by interaction with the immunophilin domain of FKBP38, including the N-terminal glutamate rich region and with a second site downstream of the TPR domain of FKBP38 [9, 10]. Our structure shows that the conformation of the side-chain of Arg 184 is partly defined (the last visible atom being the c**δ** atom of arginine), but none-the-less access to the PPIase pocket is likely to remain restricted. For example, in an FKBP12 structure (PDB 1NSG) the equivalent atom is His 87 and lies 4.6 Å from the nearest atom of the bound rapamycin [40]. In contrast, the cδ atom of Arg 184 is 3.1 Å to the exact same atom of rapamycin, when the FKBP12 structure is superimposed over that of FKBP^92-380^. Considering the rest of the Arg 184 atoms must protrude, at least partly, towards the bound rapamycin molecule, then it appears that access to the binding pocket of this PPIase domain would most likely be restricted.

#### FKBP38-DTEMEEVD peptide interactions

The structure of the FKBP^92-380^-Hsp90 peptide shows that the C-terminal carboxylate group and the C-terminal aspartate side-chain of the Hsp90 peptide are involved in a series of hydrogen bonds that is reminiscent of the carboxylate clamp seen in the MEEVD-HOP complex (**Fig 6**). The C-terminal carboxylic acid makes direct hydrogen bonds to the amine nitrogen of Asn 229 and Asn 277 and a salt bridge interaction to one of the amine nitrogen atoms of Arg 225, as well as water mediated interactions to the amine group of Lys 273 (**Fig 6**). The main-chain amide group of the terminal aspartic acid residue is also hydrogen bonded to the carboxyl group of Asn 277. The aspartate group of the peptide makes a salt bridge interaction with the amine group of Lys 307 and a series of water mediated interactions with the amide groups of Asn 305, Asn 276 and Arg 311 (**Fig 6**). The other amine group of Arg 311 is directly bonded to the main-chain carbonyl of the last glutamate residue of the Hsp90 peptide (DTEMEEVD).

For the peptide valine, the main-chain amide group forms water mediated interactions to the carbonyl and amine side-chain groups of Gln 233 (**Fig 6**). The side chain of the peptide valine is itself bound in a hydrophobic environment formed by the side chains of Asn 229, Tyr 232, Glu 233, and Asn 277. While these are polar or charged amino acid residues, the aliphatic sections of their side-chains conspire to form the hydrophobic pocket. The penultimate glutamate of the peptide (DTEMEEVD) forms a salt bridge and a water mediated interaction with the amine group of Lys 314. The remaining interactions consist of hydrogen bonds between the main-chain peptide to the side-chain carbonyl, amide or hydroxyl groups, as appropriate, of Ser 388, Asn 339, and Glu 366. Finally, the methionine side chain is bound in a hydrophobic pocket lined by Ile 306, Lys 307, Phe 310, Thr 341, Ile 342 and Asn 339 (**Fig 6**). Once again, for the polar or charged residues the aliphatic sections of the side chain conspire to define the hydrophobic pocket.

#### The carboxylate clamp is required for Hsp90 binding

The binding of the conserved MEEVD motif of Hsp90 is dependent on the carboxylate clamp interactions previously identified for this type of TPR domain [26]. Although the precise details of the carboxylate clamp interactions can vary, the interactions seen in the FKBP^92-380^-DTEMEEVD complex are none-the-less a critical component of the TPR domain interaction. To support this, we mutated Lys 307 to glutamate, which compromised the carboxylate clamp binding interaction. Using ITC, we showed that this mutant failed to bind the C-terminal domain of yeast Hsp90 (**Fig 2J and 2K**). This suggests that the carboxylate clamp of this TPR domain is similarly important in the binding of the conserved MEEVD motif of Hsp90 (**Fig 6**).

### FKBP8 does not affect the ATPase activity of Hsp90

**Fig 7:**
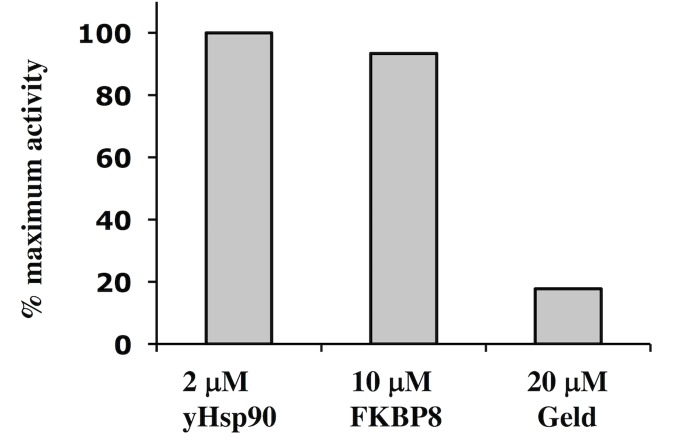
ATPase activity of yeast Hsp90. Using a 5-molar excess of FKBP^92-380,^ over Hsp90 did not substantially alter the ATPase activity of the chaperone. Geld, geldanamycin.

Previous studies showed that the peptidyl-propyl isomerase, Cpr6, could weakly activate and that the TPR domain protein Sti1 (HOP in humans) could inhibit the ATPase activity of yeast Hsp90 [41, 42]. In contrast, ATPase assays using yeast Hsp90 and FKBP8 did not show any substantial effect on the activity of Hsp90 (**Fig 7).**

## Discussion

TPR domains are versatile protein interaction modules that have been found to bind the conserved MEEVD, IEEVD EDDVE motifs of Hsp90, Hsp70 and TOMM20, respectively. FKBP proteins represent a large group of proteins that can interact with Hsp90 using TPR domains [37]. FKBPs are involved in receptor signaling, protein folding, trafficking and transcription [43]. Here we have determined the structure of FKBP^92-380^, and shown that this TPR domain is specific for Hsp90 binding over Hsp70. Specificity and high affinity binding to Hsp90 appears to include residues outside the terminal ten amino-acid residues of Hsp90 (EDASRMEEVD). While further structural work will be required to understand the complete interaction between FKBP8 and Hsp90, current attempts to crystallize larger C-terminal domain fragments of Hsp90 with TPR domains have so far failed to yield diffracting crystals.

Yeast Hsp90 C-terminal domain failed to bind the K307E mutant of FKBP^92-380^ suggesting that its binding was completely disrupted. As with other TPR domains, Lys 307 is part of an extended hydrogen bond network called the carboxylate clamp, and is critical for the binding of MEEVD containing peptides [22, 23, 27]. A similar peptide form Hsp70 (GSGPTIEEVD) to that of Hsp90 (EDASRMEEVD), which shares a substantial portion of similar interacting residues (EEVD), bound weakly to the TPR domain of FKBP8. However, intact full-length Hsp70 failed to interact suggesting that FKBP8 is specific for Hsp90, and that the structural features of the TPR domain of FKBP8 are most similar to those of Tah1[22].

The TPR and PPIase domain of FKBP^92-380^ are similar to immunophilin proteins such as FKBP52 [39]. The FKBP^1-380^ bound MEEVD containing peptides of Hsp90 were found bound in a similar conformation to that seen in other TPR domain containing proteins such as AIP, Tah1, and CHIP [22–24]. Our analysis showed that MEEVD containing peptides seen bound in the FKBP52 structure were surprisingly in an opposite polarity to that seen in this and other similar TPR domains [22, 23, 39]. In fact, the peptide conformation adopted in complex with FKBP52 varied between molecules in the unit cell of that structure (**Fig 5**) [39]. This suggested that either the peptides failed to bind correctly or were modelled incorrectly into the electron density maps. Furthermore, the hydrophobic residues, methionine and valine, of the MEEVD peptide, were unexpectedly exposed to solvent in the FKBP52 structure, which has not been previously observed, as far as we are aware. Taken together, our results suggest that the bound conformation seen in the FKBP52 structure is not physiological. In contrast, the methionine and valine residues of MEEVD in our FKBP^92-380^ structure were bound within hydrophobic pockets as seen in other similar TPR domain structures, suggesting that the conformation seen in these structures is the correct physiological state [22–24].

The regulation of the ATPase activity is a critical feature of the Hsp90 chaperone cycle which involves cycling between an open and closed state by dimerization of the N-terminal domains of Hsp90 [44–46]. Sti1 (HOP in humans) acts as a strong inhibitor of Hsp90 ATPase activity [42], thus halting the cycle, whereas the yeast peptidyl-propyl isomerase, Cpr6, weakly activates and therefore accelerates this cycle [41]. In contrast, we show that FKBP^1-380^ did not affect the ATPase activity of Hsp90. In conclusion, we show that FKBP8 specifically binds Hsp90 over Hsp70, but doesn’t appear to regulate the ATPase activity of Hsp90.
